# Diagnostic Management and Surgical Treatment of Isolated Tricuspid Regurgitation

**DOI:** 10.1155/2021/9928811

**Published:** 2021-09-10

**Authors:** Arthur Cicupira Rodrigues de Assis, Gustavo Andre Boeing Boros, Lea Maria Macruz Ferreira Demarchi, Thiago Luis Scudeler, Paulo Cury Rezende

**Affiliations:** ^1^MASS Research Unit, Instituto do Coração (InCor), Hospital das Clínicas da Faculdade de Medicina da Universidade de São Paulo, São Paulo, Brazil; ^2^Pathology Department, Instituto do Coração (InCor), Hospital das Clínicas da Faculdade de Medicina da Universidade de São Paulo, São Paulo, Brazil

## Abstract

Severe tricuspid regurgitation is especially caused by pulmonary hypertension. Primary tricuspid regurgitation in the absence of pulmonary hypertension and of unknown etiology is a very rare condition with scarce data about its diagnosis, treatment, and follow-up. The particularities of surgery indication and outcomes are still not clearly known. A 72-year-old woman with a medical history of coronary artery bypass grafting three years ago presented with shortness of breath and low limb edema. Physical examination revealed a prominent bilateral jugular turgescence, hepatomegaly, peripheral edema, and a left midsternal border holosystolic murmur, suggestive of tricuspid regurgitation. The echocardiogram confirmed the diagnosis and showed preserved right and left ventricular dimensions and function. Coronary angiography showed no new obstructive lesions and patent surgical grafts. Right cardiac catheterization revealed mild pulmonary hypertension and increased right atrium pressure. Cardiac magnetic resonance showed mild right ventricular dilation with normal systolic function and normal left chambers. No late gadolinium enhancement was detected. Because of persistent symptoms, even after optimization of medical therapy, the patient was submitted to tricuspid valve replacement surgery. Immediately after the surgery, the patient developed significant right ventricular dysfunction, with the need of continuous hemodynamic support. She had progressive clinical recovery that was confirmed by serial echocardiograms that showed improvement in right ventricular volume and function. The patient was discharged with no signs or symptoms of right heart failure. The histopathological examination showed significant and diffuse myxomatous degeneration of the leaflets. No signs of infection or vegetation nor disruption of strands were observed. This report illustrates a very rare case of symptomatic primary isolated severe tricuspid regurgitation caused by myxomatous degeneration of the leaflets. The thoroughly diagnostic workup is presented, and only the histopathological analysis of the leaflets revealed the etiologic process. Surgical treatment indicated before the onset of right ventricular failure was essential to patient's full recovery.

## 1. Introduction

The major etiology of tricuspid regurgitation (TR) is secondary to pulmonary hypertension, but primary disease occurs in about 10% of the cases [1]. In this setting, congenital and acquired diseases should be considered, such as Ebstein's anomaly, atrioventricular septal defects, myxomatous prolapse, endocarditis, rheumatic disease, carcinoid syndromes, and blunt trauma. Secondary TR is most related to right ventricular remodeling in pulmonary hypertension. Interestingly, atrial fibrillation (AF) may be considered the cause of tricuspid insufficiency rather than the consequence, especially when it is long lasting. In the acute setting, right ventricular infarction can result in papillary muscle damage and subsequent acute atrioventricular valvar regurgitation, although mitral valve is the most common.

Recently, some authors have identified that the incidence of isolated TR is increasing [1, 2]. This is mainly explained because of the use of implantable right cardiac devices and increased AF prevalence. Rarer are the cases when no causes are identified. The diagnostic management in such cases and the appropriate treatment are also challenging. Although the surgical treatment with the substitution of the impaired native valve by a prosthesis is a definitive therapeutic option, there are few data about the diagnostic workup and outcomes in such patients. Challenges are both in the acute phase after surgery and in the long-term follow-up. In this case report, the authors present one patient with stable multivessel coronary artery disease who developed symptoms of right heart failure and was diagnosed with severe isolated tricuspid regurgitation. The diagnostic workup and the surgical substitution of the tricuspid valve by a bioprosthesis are reported and discussed.

## 2. Case Presentation

A 72-year-old woman was evaluated for newly diagnosed symptomatic right heart failure, with preserved left ventricular ejection fraction and no history of precipitating factors. She complained of progressive dyspnea, orthopnea, and paroxysmal nocturnal dyspnea in the last 5 months. Prominent bilateral jugular turgescence, moderate hepatomegaly and ascites, and peripheral edema were present. Cardiac auscultation revealed an irregular rhythm and a left midsternal border holosystolic murmur, suggestive of TR.

The patient's medical history included coronary artery bypass grafting (CABG), diabetes mellitus, and dyslipidemia. CABG was performed on May 20, 2014, elective, with cardiopulmonary bypass (CPB). CPB duration was 117 minutes, and anoxia time was 70 minutes. Revascularization was anatomically complete and included the following grafts: left internal mammary artery to the anterior descending coronary artery, saphenous vein to the ramus diagonalis of the left main coronary artery, saphenous vein to the inferior interventricular artery, and saphenous vein to the second obtuse marginal of the circumflex coronary artery. There were no complications in the entire perioperative period. She remained asymptomatic during all outpatient visits every 6 months, with no clinical signs of heart failure, valvular insufficiency, or atrial fibrillation. Five months before this outpatient visit, she started with symptoms and signs of right heart failure, three years after CABG, on May 22, 2017. There was no history of previous arrhythmias. No history of fever, diaphoresis, weight loss, or trauma was reported.

The electrocardiogram showed newly diagnosed AF. Transthoracic echocardiography revealed significant enlargement of the right atrium, preserved right and left ventricular dimensions and function, and severe TR (an additional movie file shows this in more detail (see Additional file 1 at https://drive.google.com/file/d/1dI5rywG2qCX7lSrrmFuC1_IPkNvy6p1H/view)). No signs of tricuspid or mitral prolapse were observed. Coronary angiography showed no new obstructive lesions compared to previous exams and patent surgical grafts. Right cardiac catheterization revealed mild pulmonary hypertension (mean artery pulmonary pressure 33 mmHg), along with increased right atrium pressure (21 mmHg). Cardiac magnetic resonance showed the right atrium diameter and volume severely increased (antero-posterior diameter was 63 mm, and area was 27cm^2^) and mild right ventricular dilation along with normal systolic function; the tricuspid valve annulus diameter was not enlarged (30 mm in diastole); left chambers were unremarkable (Additional file 1). Subjective analysis was compatible with severe TR; no late gadolinium enhancement was detected. Additional investigation of rheumatological and oncological diseases revealed no etiological diagnosis.

The patient developed limiting symptoms of dyspnea, even after optimization of diuretic therapy, and was admitted at our hospital for surgery eleven months after symptom onset and six months after starting the optimization of medical therapy. A tricuspid valve replacement surgery was performed with a bovine pericardial prosthesis (Braile Biomedical®), with the use of cardioplegia and CPB. CPB duration was 60 minutes, and anoxia time was 34 minutes. No surgical intervention for atrial fibrillation (such as Maze procedure) was performed in order to do not prolong the intervention. Immediately after CPB, the patient developed significant right ventricular dysfunction. Hemodynamic support with aggressive preload management with high doses of intravenous diuretics, vasoactive drugs, and nitric oxide was introduced to control right ventricular failure. Serial transthoracic echocardiograms showed progressive improvement in the right ventricular volume and function, achieving normal parameters 1 week after the procedure. Analysis of the bioprosthetic valve showed no signs of dysfunction. The patient was weaned from mechanical ventilation after 48 hours and remained in the intensive care unit (ICU) for 10 days. In the ICU, she developed acute kidney injury in the first 72 hours after surgery that progressively recovered with the improvement in right cardiac function.

Despite a long hospital stay due to deep wound infection, treated with large-spectrum antibiotics and daily care by wound-infection specialized nurses, the patient showed progressive clinical recovery. The paroxysmal atrial fibrillation was treated with antiarrhythmics and anticoagulation.

In the follow-up visits, she presented well and had no signs or symptoms of right heart failure. Antiarrhythmics were progressively withdrawn, as well as anticoagulation because the patient sustained sinus rhythm after many months of follow-up.

**Figure 1:**
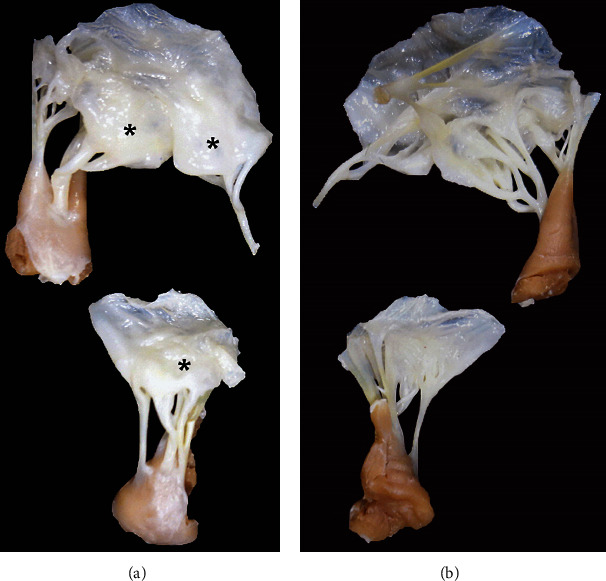
Surgical specimen from tricuspid valve repair. (a) Atrial aspect: cusps are glistening and dome-shaped (^∗^). (b) Ventricular aspect: chordae are irregularly thickened and branched.

**Figure 2:**
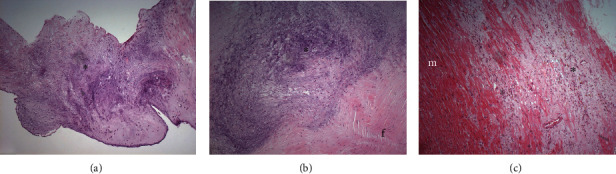
Photomicrographs from tricuspid valve. (a, b) Myxomatous degeneration (^∗^) and fibrosis (f) of the spongiosa layer. (c) Healed infarct (^∗^) in papillary muscle (m).

The anatomical study of the native tricuspid valve showed glistening and dome-shaped cusps (Figure 1(a)) and irregularly thickened and branched chordae (Figure 1(b)). The histopathological examination of the tricuspid valve showed no signs of infection or vegetation nor disruption of strands (Figure 2(a)). A significant and diffuse mixomatous degeneration was observed in the leaflets (Figure 2(b)). A very small healed infarct in papillary muscle of unspecified origin was observed (Figure 2(c)).

## 3. Discussion and Conclusions

The incidence of isolated severe TR is increasing, because of the higher prevalence of AF and the growing indication of cardiac devices [1, 2]. However, there are still no specific guidelines about diagnostic approach and management. The patient showed severe symptoms and signs of right heart failure, but no other organ impairment, such as renal or hepatic dysfunction, and this contributed to the good evolution. After confirmation of TR, an extensive investigational approach was performed. We ruled out moderate to severe pulmonary hypertension, long-term arrhythmias, and ischemic, rheumatologic, and storage diseases. The presence of AF was considered a result of severe regurgitation with subsequent right atrial enlargement and remodeling, since there was no history or documentation of arrhythmias in the regular follow-up started in 2013. Moreover, CMR showed the tricuspid valve annulus not to be enlarged. This finding strongly suggests that AF was not the cause of severe TR in this case, because echocardiographic studies of severe TR of unknown etiology in patients with AF have identified extreme annular dilation as the driving mechanism [3], suggesting, thus, that AF may be a cause rather than the consequence in such cases. Finally, the histopathological examination of the valve excluded the diagnosis of endocarditis and confirmed the myxomatous degeneration of the leaflets as the etiology of the severe TR.

Myxomatous degeneration especially affects the mitral valve apparatus, causing the prolapse of mitral valve cusps. About 30 to 40% of these patients may also have a prolapse of the tricuspid valve [4]. However, in this patient, no involvement of the mitral valve was detected, and no prolapse of tricuspid or mitral valves were observed by echocardiograms or cardiac magnetic resonance.

The main contemporary guidelines supported the approach of this case with good recommendation classes, but low levels of evidence (class I, level of evidence C, according to the European Society of Cardiology [5]; class IIa, level of evidence C, according to American Heart Association/American College of Cardiology [6]). Surgical treatment for severe TR is mainly signaled if simultaneous left valve treatment is indicated. Low levels of evidence are justified by the paucity of surgeries in this setting [7]. Studies evaluating mortality are retrospective and describe mortality of 2 to 20% in 30 days after surgery [1]. Our review of literature found one retrospective study comparing surgical and drug treatments that demonstrated a nonstatistical mortality benefit in the surgical group in 10-year survival analysis [8].

The option for the replacement instead of the repair of the valve apparatus was discussed with the surgical team before the procedure. Because of anatomical characteristics that were not favorable to repair, and in order to do not prolong CPB and surgery times, replacement was the option for this patient.

Although our patient had only preoperative mild dilation of the right ventricle with normal ejection fraction and no evidence of severe pulmonary hypertension, acute right ventricular failure occurred after cardiopulmonary bypass. The acute increase in the right ventricular afterload with the surgical resolution of tricuspid regurgitation is often poorly tolerated by the right ventricle that can evolve to severe dysfunction and cardiogenic shock right after the surgery. In this setting, aggressive diuretic therapy, inotropes, and nitric oxide were essential to the progressive improvement of right ventricular function.

In conclusion, the authors report a very rare case of severe isolated tricuspid regurgitation, from the clinical presentation, investigative process, surgical treatment, and outcomes. A thoroughly diagnostic workup associated with an early indication for surgery before the development of severe right ventricular dysfunction contributed to the good clinical evolution of the patient.

## Data Availability

Data can be available under request of the editor of the journal.

## Abbreviations

AF: Atrial fibrillation
CMR: Cardiac magnetic resonance
CPB: Cardiopulmonary bypass
TR: Tricuspid regurgitation.

## Supplementary Materials

Supplementary Materials Cardiac magnetic resonance shows severe tricuspid regurgitation; the right atrium diameter and volume severely increased and mild right ventricular dilation with normal systolic function; the tricuspid valve annulus diameter was not enlarged; left chambers were unremarkable. Transthoracic echocardiography revealed significant enlargement of the right atrium, preserved right and left ventricular dimensions and function, and severe tricuspid regurgitation.

## References

[1] Fender E. A., Zack C. J., Nishimura R. A. (2018). Isolated tricuspid regurgitation: outcomes and therapeutic interventions. *Heart*.

[2] Topilsky Y., Nkomo V. T., Vatury O. (2014). Clinical outcome of isolated tricuspid regurgitation. *JACC: Cardiovascular Imaging*.

[3] Utsunomiya H., Itabashi Y., Mihara H. (2017). Functional tricuspid regurgitation caused by chronic atrial fibrillation: a real-time 3-dimensional transesophageal echocardiography study. *Circulation. Cardiovascular Imaging*.

[4] Shah P. M., Raney A. A. (2008). Tricuspid valve disease. *Current Problems in Cardiology*.

[5] Joint Task Force on the Management of Valvular Heart Disease of the European Society of Cardiology (ESC), European Association for Cardio-Thoracic Surgery (EACTS), Vahanian A. (2012). Guidelines on the management of valvular heart disease (version 2012). *European Heart Journal*.

[6] Nishimura R. A., Otto C. M., Bonow R. O. (2014). 2014 AHA/ACC guideline for the management of patients with valvular heart disease: a report of the American College of Cardiology/American Heart Association Task Force on Practice Guidelines. *Journal of the American College of Cardiology*.

[7] Vassileva C. M., Shabosky J., Boley T., Markwell S., Hazelrigg S. (2012). Tricuspid valve surgery: the past 10 years from the Nationwide Inpatient Sample (NIS) database. *The Journal of Thoracic and Cardiovascular Surgery*.

[8] Lee J. W., Song J. M., Park J. P., Lee J. W., Kang D. H., Song J. K. (2010). Long-term prognosis of isolated significant tricuspid regurgitation. *Circulation Journal*.

