# Supplementary material for: Diagnostic Management and Surgical Treatment of Isolated Tricuspid Regurgitation
Source: Case Rep Cardiol. 2021 Sep 10;2021:9928811. doi: 10.1155/2021/9928811 (PMC8452416; doi:10.1155/2021/9928811)
Supplement: Supplementary Materials — Cardiac magnetic resonance shows severe tricuspid regurgitation; the right atrium diameter and volume severely increased and mild right ventricular dilation with normal systolic function; the tricuspid valve annulus diameter was not enlarged; left chambers were unremarkable. Transthoracic echocardiography revealed significant enlargement of the right atrium, preserved right and left ventricular dimensions and function, and severe tricuspid regurgitation. [file 9928811.f1.docx]

Supplementary file;

Cardiac magnetic resonance shows severe tricuspid regurgitation, the right atrium diameter and volume severely increased, and mild right ventricular dilation with normal systolic function; the tricuspid valve annulus diameter was not enlarged; left chambers were unremarkable.

Transthoracic echocardiography revealed significant enlargement of the right atrium, preserved right and left ventricular dimensions and function, and severe tricuspid regurgitation.

[see Additional file 1 at [https://drive.google.com/file/d/1dI5rywG2qCX7lSrrmFuC1_IPkNvy6p1H/view](https://na01.safelinks.protection.outlook.com/?url=https%3A%2F%2Fdrive.google.com%2Ffile%2Fd%2F1dI5rywG2qCX7lSrrmFuC1_IPkNvy6p1H%2Fview&data=04%7C01%7C%7C2d40cddbded04c6d694608d9676dd0af%7C84df9e7fe9f640afb435aaaaaaaaaaaa%7C1%7C0%7C637654543611694820%7CUnknown%7CTWFpbGZsb3d8eyJWIjoiMC4wLjAwMDAiLCJQIjoiV2luMzIiLCJBTiI6Ik1haWwiLCJXVCI6Mn0%3D%7C1000&sdata=U%2Byzeiw9v3uMWRSxbQIS20IHFoeozy8ABAsDMF7P85s%3D&reserved=0)]).
